# SNOMED CT in a language isolate: an algorithm for a semiautomatic translation

**DOI:** 10.1186/1472-6947-15-S2-S5

**Published:** 2015-06-15

**Authors:** Olatz Perez-de-Viñaspre, Maite Oronoz

**Affiliations:** 1 IXA NLP Group, University of the Basque Country UPV/EHU, Manuel Lardizabal 1, 20018 Donostia-San Sebastián, Spain

**Keywords:** SNOMED CT translation, Basque Language Isolate, Natural Language Processing, Finite State Transducers

## Abstract

**Background:**

The ***S**ystematized **N**omenclature of **Med**icine - **C**linical **T**erms *(SNOMED CT) is officially released in English and Spanish. In the Basque Autonomous Community two languages, Spanish and Basque, are official. The first attempt to semi-automatically translate the SNOMED CT terminology content to Basque, a less resourced language is presented in this paper.

**Methods:**

A translation algorithm that has its basis in Natural Language Processing methods has been designed and partially implemented. The algorithm comprises four phases from which the first two have been implemented and quantitatively evaluated.

**Results:**

Results are promising as we obtained the equivalents in Basque of 21.41% of the disorder terms of the English SNOMED CT release. As the methods developed are focused on that hierarchy, the results in other hierarchies are lower (12.57% for body structure descriptions, 8.80% for findings and 3% for procedures).

**Conclusions:**

We are in the way to reach two of our objectives when translating SNOMED CT to Basque: to use our language to access rich multilingual resources and to strengthen the use of the Basque language in the biomedical area.

## Introduction

SNOMED Clinical Terms (SNOMED CT) [1] is widely recognized as the most comprehensive, multilingual clinical health-care lexicon. By using SNOMED CT in electronic health records the consistency of the representation improves, benefiting in this way individuals [2].

The two co-official languages in the Basque Autonomous Community, Spanish and Basque, should be used in Osakidetza (the Basque Sanitary System). Even though in Osakidetza the two languages are used, Spanish is a much stronger language and Basque is hardly used in the documentation services. In 2005 Osakidetza approved its first *Basque Scheme to normalize the use of the Basque language in Osakidetza *for the period 2005-2012. In the evaluation of this plan [3] they concluded that the greatest progress in the use of Basque was done in the area of language profiles (accreditation of language profiles, jobs with mandatory Basque knowledge etc.). For the second scheme (period 2013-2019) [3] one of the hubs that needs to be strengthened is the use of Basque in the documents: "emphasis should be placed on the documents of a care nature through the normalizing and systematizing of bilingual models of documents, bearing in mind that their adaptation or production in Basque must be facilitated and simplified by professionals. In parallel, in order to have bilingual clinical records available, an in-depth study must be started without further delay and the aspects that influence the process to create and exploit information must be analyzed". However, writing bilingual clinical records can be a tedious work for doctors and what is more, a misuse of their time. The alternative solution, the translation of the records by professional translators could be very expensive.

As far as we know, in other bilingual countries like Canada, the communication language between patient and doctor is established on demand by the patient [4]. This is not possible in our scenario, as some doctors are not able to understand Basque. As the safety of the patients cannot be put at risk, the comprehension of previous or current clinical records is essential for every health professional. Therefore, Spanish is the only language used nowadays for documentation. But this fact produces a complex scenario in which Basque language is isolated as a merely oral interaction tool, and doctors develop the ability to translate from verbal communication in Basque to written notes in Spanish. Nowadays, patients do not have the option of having their medical records in Basque. In a normalized scenario, the clinical notes would be written in the preferred language set by patient-doctor communication.

We agree with the statement that "the summarized clinical history of any patient should be at least in the two co-official languages to assure the security of their assistance". This statement has been made by an Osakidetza committee that has the objective of giving recommendations and analyze whether a bilingual clinical records system is possible or not. In this context, a multilingual version of SNOMED CT including Basque will help to produce such bilingual (or even multilingual) clinical records. That is, by means of a "text to SNOMED" matching tool and a multilingual terminology service based on SNOMED CT, we designed a prototype to help doctors writing clinical records in Basque. By means of a fast and easy disambiguation process of the most relevant medical terms in the record written in Basque, the prototype produces in the present stage of development a minimal Spanish version of the terminological content. The prototype also incorporates a spell-checker adapted to the medical domain based on the specialized terminology from the biomedical domain. The prototype is still in a very early phase and the Basque SNOMED CT terminological content must be completed and manually checked, but it shows i) a use case for the work we present in this paper and, ii) that the creation of a tool to help in the writing of medical records in Basque is feasible. We have presented this prototype to the committee mentioned before with a very positive feedback. The decision about writing medical records in Basque is out of the scope of the scientific discussion and will be taken by the mentioned committee.

In conclusion, one of our goals in this work is to try to enforce the use of Basque in the biomedical area by offering to the medical personnel a standard medical terminology and thus, to safeguard patients and doctors linguistic rights. As mentioned, another goal is to attain multilingual medical resources in the Basque language. These objectives can be reached, in our opinion, by semi-automatically translating the terminology content of SNOMED CT. We will focus on the most populated SNOMED CT hierarchies.

To translate the terminological content of SNOMED CT, we have defined a four phase algorithm that is based on Natural Language Processing (NLP) techniques and that is presented in [6]. In that paper we outlined the main ideas of the translation algorithm and the implementation of the first two phases (out of four) as well as the Phase 0 about the mapping between SNOMED CT and ICD-10. In the current paper we extend the explanation and we improve the base system. We also expose some new experiments and the corresponding results.

Multilingual lexical resources are the source of information in the implementation of the first phase of the algorithm, while a finite-state approach that uses medical affixes together with transcription rules in order to obtain clinical terms in Basque, is used in the second phase. In both approaches, we use mainly English as source language and in the first phase we also used Spanish-Basque dictionaries to complement the information sources available.

Regarding the third phase which aim is the translation of complex terms, we are analyzing their nature in the English version of SNOMED CT and we found out that many different terms share a specific structure. In Table 1 we show some of the most obvious structures or patterns found from shallow experiments. For instance, there are 1,498 terms with the structure ''[PHARMPRODUCT|SUBSTANCE] + allergy'', that is, a pharmacological product or a substance followed by "allergy", like "urokinase allergy", "cortisone allergy" or "phentolamine allergy". Our hypothesis is based on the evidence that we have already the translations of some chunks within the complex term. In this step the translation application should generate the Basque equivalences using the already translated components and some generation rules.

**Table 1:** Structures of the SNOMED CT terminology content.

Pattern found	Quantity
[PHARMPRODUCT|SUBSTANCE]+allergy	1,498
[PHARMPRODUCT|SUBSTANCE]+adverse+reaction	1,488
[PHARMPRODUCT|SUBSTANCE]+poisoning	847
[PHARMPRODUCT|SUBSTANCE]+overdose	567
[PHARMPRODUCT|SUBSTANCE]+poisoning+of+undetermined+intent	432
intentional+[PHARMPRODUCT|SUBSTANCE]+poisoning	429
accidental+[PHARMPRODUCT|SUBSTANCE]+poisoning	428
...	...

The fourth and last step will adapt a rule-based automatic translation system called *Matxin *[24] to the medical domain.

Issues as i) the design and implementation of the translation application, ii) the way we manage the terminology and, iii) the representation of the terminological content as meta-data (knowledge representation), are not addressed in this paper. Term generation is the main subject of this paper. The translation software framework we use to manage the terms is alredy developed and operative. The schema for knowledge representation is designed and is also in use [5].

The current article is an extended version of the work published in *The Fifth International Workshop on Health Text Mining and Information Analysis (Louhi 2014) *conference [6]. The main novel aspects are, i) an extended introduction and motivation of the work exposed, ii) the inclusion of Spanish-Basque lexical resources, iii) a detailed explanation about new approaches developed for generating simple Basque terms, iv) a detailed description of the finite-state transducers used in the algorithm, v) a more detailed evaluation of the phases already developed of the algorithm and, vi) a table quantifying the number of concepts from SNOMED CT in each of the hierarchies and semantic classes (English and Spanish versions).

The remainder of this paper is arranged as follows: first, a Background section where we justify the work and relate it to other SNOMED CT translations. In the Methods section we focus on the implementation of the first two phases of the translation algorithm. Finally, Results are presented and discussed, and the Conclusions and future lines of this work are listed.

## Background

"Basque language, also called Euskara or Euskera, language isolate, the only remnant of the languages spoken in south-western Europe before the region was Romanized in the 2nd through 1st century BCE. The Basque language is predominantly used in an area comprising approximately 3,900 square miles (10,000 square kilometres) in Spain and France"[7]. It is spoken in the Basque Country, a region placed in the northeastern part of Spain and in the southwestern part of France. Basque is a minority language that persists between Spanish and French, two powerful languages. Today Basque is in its standardization process and holds co-official language status in the Basque Autonomous Community but during centuries it was excluded from educational systems, media, and industrial environments. Nowadays, in the Basque Autonomy Community 36.4% of the population knows and uses well the Basque language (30 years ago was 22%); 19.3% is Basque receiver, that is, this percentage of the population understands and reads the language but cannot write or speak it; and, 44.3% do not know the language (30 years ago two thirds of the population was in this situation). There are 749,182 Basque speakers, 318,000 more than in 1981. That is, as mentioned in the *fifth sociolinguistic map of the Basque Autonomous Community *[8] the number of Basque speakers has increased in the working world and the age-range where it has increased the most is in people that are less than 20 years old. Even though the data shows that the use of Basque is increasing, primarily between young people, these people are not in the labor market yet. Due to all these characteristics, the Basque Language in the health system has very low use. With this work we aim at facilitating the use of the Basque language in the biomedical area.

In SNOMED CT concepts are linked to terms in different languages by means of concept identifiers, which makes of SNOMED CT a multilingual resource. With a Basque version of SNOMED CT, we can obtain the terms in our language linked to terms in all the languages represented in SNOMED CT. Besides, SNOMED CT is part of the Metathesaurus of UMLS (Unified Medical Language System [9]), so other lexical medical resources containing SNOMED CT concepts (RxNorm, MeSH...) can be accessed by Basque speakers.

SNOMED CT has been widely used with commercial as well as research purposes. In 2006 a survey on health information technology (HIT) vendors was carried out [10] in order to study the predominance of SNOMED CT in electronic medical records (EHRs). The authors of the study concluded that the respondents who were already working with SNOMED CT increased its use in EHRs for clinical decision support, encoding of health-care data, health information exchange and patient assessment. Posterior surveys [11] on vendors indicated that although SNOMED CT is highly used in production systems, most of these uses are elementary and do not benefit from the rich semantics of the terminology. In the *SNOMED in Action *initiative [12] several uses of this terminology are listed. Among others it is used to document diagnoses and problems for ambulatory clinic patients, evidence-based medicine, and so on.

One of the strengths of SNOMED CT is its nature as a standard. As it is pointed in [13] "it's software aimed at eliminating potentially dangerous misunderstandings over what medical terms actually mean to different clinicians, researchers, and even to patients". In [14] how SNOMED CT is implemented in 12 health-care organizations across eight countries was studied by means of a survey that took into account design, use and maintenance issues. After this survey they described the advantages of using SNOMED CT as i) clinicians can record the exact diagnosis making use of the large number of synonyms available, ii) via SNOMED CT International Classification of Diseases (ICD) codes can very easily be generated, iii) SNOMED CT offers clinicians the best coverage to describe their use cases and, iv) its standard nature makes patients' records legible.

The paper entitled "The need for SNOMED CT translations" [15] aims at promoting "a discussion about the European wide availability of language-specific SNOMED CT translations" because the authors think that "Language-specific translations of SNOMED CT are necessary for bringing value-added applications into clinical routine in non-English speaking countries". The authors of the paper recommend the introduction of SNOMED CT across Europe, with special emphasis on German as the largest language group. We agree with the introduction of SNOMED CT but we want to remark that also minority languages should be considered if they want to survive, which is one of the reasons why we are interested in working with Basque.

"Today, SNOMED CT is available in US English, UK English, Spanish, Danish and Swedish. Translations into French, Lithuanian, and several other languages are currently taking place" [16]. As referenced in the IHTSDO web-page, the translation of SNOMED CT to other languages has been already performed using different techniques. These translations were done using exclusively automatic translation helping systems (this is the case of French [19]), combining automatic translation and manual work (that is the case of Chinese [18]), or manually (in Danish language for example [17]). In [20], three kinds of translations from English to German of a set of 500 SNOMED CT terms are compared: i) one translation was performed by professional medical translators, ii) another one used Google Translate [21] and, finally iii) medical students translated the same group of terms. They concluded that machine translation and the employment of student translators are considerable alternatives with "surprisingly" good results, but these methods are not acceptable for the production of terminological standards. However, the authors think that "the combination of machine-translated text with subsequent post-editing by humans could be another translation strategy that reduces time and produces quality translations". This is, in fact, the approach we want to follow in this work.

The guidelines for the translation of SNOMED CT [22] recommended by the IHTSDO have been followed to design the translation task described in this paper.

Spain is a member of the IHTDSO. In May of 2014 this institution presented the *"IHTSDO Policy on Support for Member Country Translation" *proposal, which supports the translation of the CORE of SNOMED CT from English into other languages. If the institutions of the Basque Country obtained this support for the manual translation of SNOMED CT into Basque, the generated corpus of 5,000 manually translated terms would be essential for the evaluation of our system.

## Methods

To deal with the translation of SNOMED CT, two strategies can be used: i) the enrichment of the terminology in the SNOMED CT version from Spain (in Spanish) with Basque (as well as with Catalan, or Galician) value sets for the most important concepts and, ii) the creation of an independent SNOMED CT version in Basque. We decided to use the second approach for these reasons: i) We want to collect and create the most extensive terminology possible, not wasting the resources we already have (dictionaries for instance) and, ii) it facilitates the extraction of the most important concepts to enrich the Spanish version.

In this section after describing the analysis of two SNOMED CT releases that led us to choose the source SNOMED CT version for the translation task, we will describe in detail the first two phases of the algorithm as these are the ones already implemented and evaluated.

### Analysis to choose the source language in SNOMED CT

SNOMED CT is composed of almost 300,000 active concepts which are represented by descriptions or terms. This terminology corresponds to the core terminology found in electronic health records and it is organized in hierarchies. SNOMED CT terminological content offers a thorough coverage of the terms used to write the record patient conditions [23]. Concepts are defined by means of description logic axioms and are also used to group terms with the same meaning. In this paper we will refer to these descriptions as terms.

SNOMED CT divides the descriptions in three types (see Table 2): Fully Specified Names or FSN, Preferred Terms or PT and Acceptable Synonyms or Synonyms. The description used to unambiguously describe the concept is called Fully Specified Name. Those descriptions are easily identifiable as they show a semantic tag in parenthesis at the end of the description, e.g. *disorder*, that expresses its semantic category and in consequence, the hierarchy it belongs to, e.g. *Clinical finding/disorder *(even if the hierarchical structure is defined by the relationships between concepts). Regarding the terminology of clinical records, that is, proper "terms" or "descriptions", SNOMED CT distinguishes PTs and Synonyms. PTs are the most common way to name the meaning of the concept according to the IHTSDO. Synonyms are additional terms used to refer to the same concept. Thus, for each SNOMED CT Concept, a language has to define a FSN, a PT and as many Synonyms as there are used to refer that concept (it could have zero to many Synonyms).

**Table 2:** Description types in SNOMED CT for the concept: 95575002 - Obstruction of pelviureteric junction.

*Description*	Type
Obstruction of pelviureteric junction (disorder)	FSN
Obstruction of pelviureteric junction	Preferred Term
PUJ - Pelviureteric obstruction	Synonym
PUO - Pelviureteric obstruction	Synonym
Pelviureteric obstruction	Synonym
UPJ - Ureteropelvic obstruction	Synonym
Ureteropelvic obstruction	Synonym

Table 3 shows the 18 hierarchies SNOMED CT has its content divided into (plus the metadata hierarchy) and the number of FSNs in each hierarchy and language. We extracted this data from the last version released of the International Release in English, dated on 2014-01-31 and the Spanish version of the International Release, dated on 2014-04-31. As mentioned before, SNOMED CT groups its concepts in hierarchies such as *Clinical finding/disorder*, *Organism*, and so on. These hierarchies differ not only in the content, but also in the requirements for translation. For example, some hierarchies like *Organism *do not require the Preferred Term to be localized, because it corresponds to the taxonomic one. The IHTSDO offers the guidelines for the translation of SNOMED CT in [22], and it describes among others, the recommendations that are important for each hierarchy. The FSN will not be translated, but generated after the validation of the PT, following the rule of creating it by appending a semantic tag to the PT.

**Table 3:** SNOMED CT hierarchies and number of FSNs.

	English version		Spanish version	
**Hierarchy**	**Semantic Tag (ST)**	**# FSN**	**Semantic Tag (ST)**	**# FSN**

**Clinical**	**disorder**	**66,239**	trastorno	66,199
**Finding/disorder**	**finding**	**33,573**	hallazgo	33,613
**Procedure/intervention**	**procedure**	**51,149**	procedimiento	51,149
	**regime/therapy**	**2,480**	régimen/terapia	2,480
**Organism**	**organism**	**33,157**	organismo	33,157
**Body structure**	**body structure**	**24,950**	estructura corporal	24,953
	**morphologic abnormality**	**4,509**	anomalía morfológica	4,509
	**cell**	**626**	célula	626
	**cell structure**	**504**	estructura celular	501
Substance	substance	23,845	sustancia	23,845
Pharmaceutical/biologic product	product	16,759	producto	16,759
Qualifier value	qualifier value	8,944	calificador	8,944
Observable entity	observable entity	8,278	entidad observable	8,278
Event	event	3,671	evento	3,670
Situation with explicit context	situation	3,561	situación	3,561
Social context	occupation	3,852	ocupación	3,852
	person	425	persona	425
	ethnic group	262	grupo étnico	262
	religion/philosophy	203	religión/filosofía	203
	life style	21	estilo de vida	21
	social concept	23	contexto social	23
	racial group	19	grupo racial	19
Physical object	physical object	4,513	objeto físico	4,513
Specimen	specimen	1,440	espécimen	1,440
Environment or geographical location	environment	1,094	medio ambiente	1,094
	geographic location	617	localización geográfica	617
Staging and scales	assessment scale	1,077	escala de evaluación	1,077
	tumor staging	214	estadificación tumoral	214
	staging scale	16	escala de estadificación	16
Special concept	navigational concept	640	concepto para navegación	640
	namespace concept	169	espacio de nombres	169
	special concept	1	concepto especial	1
Record artifact	record artifact	224	elemento de registro	224
Physical force	physical force	171	fuerza física	171
Metadata	foundation metadata	169	metadato fundacional	169
	core metadata concept	31	metadato del núcleo	32

We analyzed the multilingual lexical resources available for Basque in the biomedical domain, and the languages in which SNOMED CT is released, and we concluded that two source languages can be used for our translation task: English and Spanish. As Basque is an isolate language, it is not related to either of the mentioned source languages. The linguistic characteristics of Basque differ greatly from those in English and Spanish, so there is no linguistic relatedness reason to choose one of these languages as translation source. Thus, we analyzed both versions of SNOMED CT to choose the best option. The versions we analyzed are dated the 31-07-2012 for English and the 31-10-2012 for Spanish and we focused on the Release Format 2 (RF2) and Snapshot distributions. We must highlight that the Spanish version of SNOMED CT is a manual translation of the English version and at that time the Spanish version was not a complete version.

Even if both languages have the same number of active concepts (296,433 concepts), the Spanish version has a significantly smaller number of terms because the version is at a preview stage: 15,715 concepts in Spanish lack PTs and Synonyms. At a first stage, this data led us to choose as the source for the translation the English version of SNOMED CT but we soon realized that we could not leave aside the already available Basque-Spanish pair resources.

In order to establish a priority between hierarchies for the translation, we counted the number of terms in each hierarchy. The most populated hierarchies both in previous and current versions are: *Clinical finding/disorder *(99,812 concepts) and *Procedure *(53,629 concepts) followed by *Organism *(33,157 concepts) and *Body Structure *(30,589 concepts). IHTSDO indicates in the translation guidelines that Preferred Terms in the *Organism *hierarchy should not be translated, so we decided to prioritize the translation of the *Clinical finding/disorder*, the *Procedure *and the *Body Structure *hierarchies.

In the next subsection we will describe deeply the first two phases of the algorithm.

### Phase 1: lexical resources

The first phase corresponding to the lexical resources has been performed for both language pairs, English-Basque and Spanish-Basque. Although, we decided to take English as source language, we cannot discard the lexical resources available for the Spanish-Basque pair. Thus, we take advantage of the robustness of the English version and of the bigger amount of lexical resources available in the Spanish-Basque pair. These are the multilingual specialized dictionaries used to obtain the Basque equivalences.

• *ZT Dictionary *[25]: a specialized dictionary of science and technology that contains areas included in SNOMED CT as medicine, biochemistry, biology... It contains 10,626 English-Basque equivalences and 10,971 Spanish-Basque equivalences.

• *Nursing Dictionary *[26]: a small dictionary of the nursing domain that has 4,155 entries in the English-Basque chapter and 4,671 entries in the Spanish-Basque one.

• *Glossary of Anatomy*: anatomical terminology used by university experts in their lectures. In its development phase it has 2,818 entries for the English-Basque pair, and 3,940 entries for the Spanish-Basque pair.

• *ICD-10 *[27]: The 10th version of the International Classification of Diseases was translated into Basque in 1996. We combined it with the Spanish and English versions and we obtained a dictionary of 6,936 equivalences between English and Basque and 8,842 equivalences between Spanish and Basque.

• *EuskalTerm *[28]: the biggest multilingual terminology bank available for Basque with 75,860 entries. Regarding the domain of biomedicine, the bank contains 32,301 term equivalences. These equivalences are all available for the Spanish-Basque pair, and 10,506 equivalences for the English-Basque pair.

• *Elhuyar Dictionary *[29,30]: a general dictionary that is available for the English-Basque pairs and Spanish-Basque pairs. The English-Basque version contains 39,164 equivalences from English to Basque and the Spanish-Basque version contains 62,215 entries.

• *Dictionary of Sanitary Administration *[31]: a small dictionary that contains 1,799 entries for the Spanish-Basque pair corresponding to the administration of the sanitary domain.

As mentioned before, *Elhuyar Dictionary *is a general dictionary that also contains some specialized terminology. Taking into account the wide variety in SNOMED CT terminology, we decided to use this general dictionary to increase the number of translation pairs only when the source term (English or Spanish) does not exist in the rest of dictionaries. Thus, we limited the big amount of ambiguous equivalent Basque terms of the biomedical domain extracted from *Elhuyar Dictionary*. The use of this dictionary provided i) equivalences of terms not directly related to the biomedical domain (e.g. terms from the "social context" or "qualifier" hierarchies), and also, ii) the equivalences of chunks for the translation of complex terms, and in consequence, the generation of new terms in Basque.

### Phase 2: finite state transducers and biomedical affixes

This subsection explains the system that obtains Basque equivalent terms from English simple terms based on Finite State Machines. This approach is based on the idea that a considerable amount of medical terms can be created as neologisms [32], that is, new words and meanings can be created by the concatenation of existing morphosemantic units. These units usually have Greek and Latin origins and their meaning is known by the specialists. In [33] the author specified that about three-fourths of the medical terminology is of Greek origin. Finite State Transducers are appropriate for dealing with the compositional structure of those medical simple terms.

First of all, we will describe the general system for the translation process. Next, we will explain the first approach developed from the baseline system [34]. Finally, we will explain the improvements proposed by experts that have been introduced in the system.

#### Baseline translation process

The generation of Basque equivalent terms from English terms is performed in three phases: first the identification of the affixes; secondly the translation of the affixes, and finally the composition of the translated affixes. All the linguistic information is stored in lexicons, and rules are written for the process of identification, translation and morphotactics.

Listing 1 shows the Finite State Transducer for the identification of the affixes. The lexica of the affixes is loaded (lines 1-6) and then any prefix (the "*" symbol indicates 0 or more times) followed by one unique suffix is identified. The connecting vowel -o- may be also identified as it is commonly used in connecting two elements of Greek origin. To mark the limits of the affixes the "+" symbol is used. The full explanation about the regular expressions used in Foma is available in [35,36].

Listing 1 Rules for affix identification

1 read lexc prefixes.lex

2 define PREFALL

3 define PREF PREFALL.u ;

4 read lexc suffixes.lex

5 define SUFALL

6 define SUFF SUFALL.u ;

7 regex [[[PREF 0:+] (o 0:+)]* SUFF] ;

In order to reduce the overproduction of the transducer, we fixed the criteria to pick the output with less identified parts. For instance, for the term "photodermatitis" four possible outputs are generated:

• photo+dermat+itis: 3

• photo+derm+at+itis: 4

• phot+o+dermat+itis: 4

• phot+o+derm+at+itis: 5

In this case, the first identification is given to the translation transducer as it contains only three parts.

Following this criterion, even though we can reduce the overproduction we cannot always avoid it. In fact, if we analyze the lexicon of the prefixes we obtain that 93% of the translation pairs are equal to the ones obtained from transcription rules that will be described in the First approach. In Example 1 we can observe how the equivalence given to "cholecyst" (*"kolezist" *in Basque) is the same as the combination of *"kole" *and *"zist" *so the translation transducer will output the same string. That is to say, in most cases the overproduction is reduced once the translation and the composition FSTs are applied as the output equivalent term will be the same.

**Example 1 **Some preffix equivalences in our lexicon.

cholecyst:kolezist #;

chole:kole #;

cyst:zist #;

The combination of the Finite State Transducers for the translation and for the composition using morphotactics is shown in Listing 2. First, the lexicons for the translation task are loaded (1-4), and then 28 rules for morphotactics are applied (simplified in the rule numbered 5). Some of these rules were determined empirically by analyzing examples from dictionaries, and others have as a basis the orthographic rules set by the Royal Academy of the Basque Language [37]. The translation rule (shown in rule number 6) is composed of the word-start mark (the ˆ symbol), the prefix (named TRANSPRE) followed by the optional linking "o" zero or more times, and a single compulsory suffix (TRANSSUF); finally in the step number 7 the transducer combines the translation (TRANS) and the morphotactic finite state transducers (MORPH) by means of a ".o." composition rule.

Listing 2 Rules for the affix translation

1 read lexc prefixes.lex

2 define TRANSPRE

3 read lexc suffixes.lex

4 define TRANSSUF

5 define MORPHO ...

6 define TRANS (ˆ) [[[TRANSPRE +] (o:o +)]* TRANSSUF] ;

7 regex TRANS .o. MORPH ;

We decided to make the suffix compulsory as we discovered that the equivalences of the suffixes are more complex than the equivalences of the prefixes. That is, only 22% of the suffixes follows the transcription rules mentioned before, and what is more, we have not been able to find a pattern based on morphotactics for those endings. Thus, we consider that for this stage of the development the suffix must be compulsory to guarantee a higher precision of the translation. Besides, this condition seems to exclude terms that do not follow a "prefix, root and/or suffix" structure which is the structure this method has been designed for. Example 2 shows the whole process with an example. First, we identify the prefixes and suffixes of the English input term by means of the transducer that marks those affixes (schiz+encephal+y). Then, we obtain the corresponding Basque equivalent for each part and we form the term (eskiz+entzefal+ia).

**Example 2 **Basque simple term generation.

**Input term: **schizencephaly

**Identified affixes: **schiz+encephal+y

**Translated affixes: ***eskiz+entzefal+ia*

**Output. Basque term: ***eskizentzefalia*

As we said before, in order to obtain a well formed Basque term, we apply different morphotactic rules. For example, in Basque, there are not words that start with "r" and an "e" is needed at the beginning. Example 3 shows a case where the translated prefix *"radio" *needs of the mentioned rule, obtaining *"erradio"*.

**Example 3 **Morphotactic rule application.

**Input term: **radionecrosis

**Identified affixes: **radio+necr+osis

**Translated affixes: ***radio+nekr+osi*

**Output. Basque term: ***erradionekrosi*

In order to identify the English medical suffixes and prefixes we have joined two lists: the "Medical Prefixes, Suffixes, and Combining Forms" from Stedman's Medical Dictionary [38] and the "List of medical roots, suffixes and prefixes" from Wikipedia [39]. From the roots we analyzed, we deduced that their behavior is similar to prefixes when it comes to the composition of words, and so we will label them and include them as prefixes. We manually generated a list of 826 prefixes and 143 suffixes with their Basque equivalents.

To perform the translation task, we manually deduced the appropriate Basque equivalents of the medical affixes. We infer the translation of the affixes from term pairs in specialized dictionaries such as *Zientzia eta Teknologiaren Hiztegi Entziklopedikoa *(Dictionary of Science and Technology) [25], Euskalterm [28] and *Erizaintzako Hiztegia *(Nursing Dictionary) [26]. Table 4 shows an example where the equivalent of the "encephal" prefix is obtained, deducing that *"entzefal" *is the most appropriate equivalent.

**Table 4:** The translation of the "encephal" prefix.

English terms	Basque terms
echo**encephal**ogram	*eko**entzefal**ograma *
**encephal**itis	***entzefal**itis*
**encephal**omyelitis	***entzefal**omielitis *
leuko**encephal**itis	*leuko**entzefal**itis*
...	...

From all the prefixes and suffixes listed, we were able to deduce 812 prefixes and 139 suffixes for Basque. They were supervised by an expert so the confidence in the equivalences is high. This technique allows the inference of new medical terms which do not appear in dictionaries.

This baseline approach gave us a precision of 0.94 and a recall of 0.52 as we show in the Results section. Even if the precision is good, the low recall forced us to improve the system, as we will show in the following section.

#### First approach

In order to improve the very low recall of the Baseline approach, we focused on increasing the number of affixes and implementing transcription rules from English/Latin/Greek to Basque.

To enrich the lexicons of the affixes we included the "Suffix Prefix Dictionary" from Macroevolution [40] and some prefixes from the "Mosby's Medical Dictionary". Thus, we obtained 1,703 prefixes and 630 suffixes manually generated and checked by an expert, and we inferred 40 rules for transcription.

In the Baseline implementation only medical terms fully identified are translated. For example, terms with the prefix "phat" are not translated as this affix does not appear in the prefixes and suffixes lexicons. In consequence, terms such as "hypophosphatemia" are not translated even though the "hypo", "phos" and "emia" affixes are identified and appear in the lexicons.

As mentioned before, 93% of the prefixes lexicon were the same as the ones created with transcription rules. Thus, we analyzed the general behavior of the "not identified parts" and prefixes lexicon in the Basque equivalent terms, and we defined 40 transcription rules. For instance, "v" is transcribed as "b" in Basque or "c" is transcribed as "z" whenever is followed by "e", "i" or "y" and is transcribed as "k" otherwise. The term "diverticulitis" has the Basque equivalent "dibertikulitis" as we find in the ZT Dictionary and in the Nursing Dictionary. This example shows how the rules behave: the "verticul" part is not in our extended lexicon, but we can observe that with the two transcriptions explained earlier we can obtain the proper Basque equivalent: "bertikul" ("v" is "b" and "c" is "k" as it is followed by "u").

In order to identify the parts that do not appear in the lexicons, we introduced a new rule for the identification task. In Listing 3, line 3 identifies the parts that do not appear in the lexicons writing the # symbol at the end of the part. Line 2 corresponds to the identification explained in Listing 1, and line 4 composes the two previous rules by a priority union (.P. is used).

Listing 3 Rules for the affix identification with parts which do not appear in the lexicons

1 ...

2 define IDEN1 [[[PREF 0:+] (o 0:+)]* SUFF] ;

3 define IDEN2 [(? + 0:# +) [PREF 0:+]]* (? + 0:# +) SUFF ;

4. regex IDEN1 .P. IDEN2 ;

We had to adapt the criterion to choose among the possible identifications. In this case, whenever a term has non-identified parts, we add to the number of parts the number of characters in the non-identified part. Thus, we choose the term with less non-identified characters, but also with the more robust identification. For example, for the term "diverticulitis" four possible identifications are given (the first number indicates the length of the non-identified part, while the second one gives the number of parts):

• diverticul#+itis: 10 + 2 = 12

• divertic#+ul+itis: 8 + 3 = 11

• di+verticul#+itis: 8 + 3 = 11

• di+vertic#+ul+itis: 6 + 4 = 10

In this case, even if the last option has more parts, it contains less non-identified characters and the addition of the two lengths results in the smallest number. Thus, it will be the one chosen.

Listing 4 shows three of the rules defined for the transcription of the parts. Lines 2 and 3 describe the rule that converts "c" into "k" or "z" depending on the following character and line 4 corresponds to the transcription of "v" into "b". Lines from 5 to 10 show the rule about the palatalization of the sibilants "z", "s" and "x". In Basque, sibilants whenever are followed by a vowel and preceded by "n", "l", "r" or "m", are palatalized by adding a "t" before them.

Listing 4 A few rules for the transcription

1 ...

2 define C c −> k | | [ noC ] [ a | o | u | noHC | #] , ,

3           c −> z | | [ noC ] [ e | i | y ] ;

4 define V v −> b ;

5 define Vow [ a | e | i | o | u | y ] ;

6 define Sib [ s | z | x ] ;

7 define PAL n −> n t | | Sib Vow , ,

8             l −> l t | | Sib Vow , ,

9             r −> r t | | Sib Vow , ,

10             m −> n t | | Sib Vow ;

11 ...

By means of these improvements, we are able to translate all the simple terms that contain just a suffix from the suffix lexicon. That is, we still keep the suffix compulsory as mentioned in the Baseline approach. We check whether the term contains any prefix from the translation pair list in order to identify the parts. After the identification, we translate the prefixes and the suffix from the translation pair list and the rest of the parts by means of transcription rules. We finally apply the morphotactic rules from the baseline system to join the translated or transliterated parts and thus create the equivalent Basque term.

Example 4 shows step by step the work carried out. In the first step we take the input term "hypophosphatemia" and we split it into the possible affix combination (in this case "hypo+phos+phat#+emia" or "hypo+phos+phat#+em+ia"). In the second step, we get the Basque equivalences of the affixes ("hipo+fos+fat+emia" or "hipo+fos+fat+em+ia"). Finally, we apply the morphotactic rules to compose the well-formed Basque term (in both cases "hipofosfatemia" is generated).

**Example 4 **Term translated by means of affix equivalences.

**Input term: **hypophosphatemia

**Identified affixes: **hypo+phos+phat+emia, hypo+phos+phat+em+ia

**Translation of the affixes**: hipo+fos+fat+emia, hipo+fos+fat+em+ia

**Morphotactics output term: ***hipofosfatemia*

With this improvement the recall of the system increases to 0.826. However, as it is often the case, the precision decreases to 0.813 as shown in the Results section. This loss in the precision led us to analyze the mistakes made by the system with several experts specialized in Basque terminology from the medical domain.

#### Second approach

Following the advice provided by the experts we restricted the criteria used to choose the terms to be eligible for translation. On the one hand, we reduced the lexicon of the suffixes, excluding the suffixes that are used in common words. That is, suffixes like "-tion" or "-able" have been excluded as they are not exclusive from the biomedical domain, and only suffixes closely related to this specialized terminology were used to conform the lexicon of suffixes. In addition, short prefixes with three characters or less were excluded from the lexicon of prefixes to eliminate prefixes that could be found within terms. For instance, the prefixes "an-" or "col-" were taken off.

In the following enumeration we list the criteria to identify the components of a term. If we cannot separate the components with the first criterion the second one is tried. If it is not applicable, the last one is attempted.

1 The whole term is identified by means of the extended lexicons (line 8 in Listing 5).

2 The term has the suffix that appears in the reduced lexicon of suffixes (line 9 in Listing 5).

3 The term has the suffix that appears in the extended lexicon of suffixes and contains at least one prefix from the reduced lexicon of prefixes (line 10 in Listing 5).

Listing 5 Rules for the affix identification second approach

1 ...

2 read lexc prefixes Reduced.lex

3 define PREFREDUCED

4 define PREFRED PREFREDUCED.u ;

5 read lexc suffixes Reduced.lex

6 define SUFFREDUCED

7 define SUFFRED SUFFREDUCED.u ;

8 define IDEN1 [[[PREF 0:\%+] (o 0:+)]* SUFF] ;

9 define IDEN2 [(? + 0:# +) [PREF 0:+ ]]* (+ 0:# +) SUFFRED ;

10 define IDEN3 [(? + 0:# +) [PREFRED 0:+ ]] + (? + 0:\ # +) SUFF ;

11 regex IDEN1 .P. IDEN2 .P. IDEN3 ;

The selection of the suffixes to be excluded has been made by consulting the suffixes in a general dictionary of English suffixes in the Wiktionary [41]. We manually checked the definition of each of the suffixes in the dictionary, so we could exclude the suffixes with a general meaning. We have also excluded the suffixes that are used in non-transcriptable terms like "-hood". For example, this suffix used in "childhood" or "manhood" have as equivalents in Basque two completely different suffixes: "-aro" in *"haurtzaro" *("childhood") and "-tasun" in *"gizontasun" *("manhood"). We are aware that the manual procedure may be prone to errors, however, we have reviewed the suffixes appearing in the general suffixes list, and so, the most common ones were excluded.

In this process we had to make certain decisions as in the case of the suffix "-on". Even if its three senses are related to biology or chemistry, it is the ending of many general suffixes as "-tion" or "-isation", being "-tion" the most popular one. As those suffixes have been excluded from the lexicon, we decided to exclude the suffix "-on", as by means of including it we will be identifying the terms with the "-tion" suffix in most of the cases.

The exclusion process led us to exclude 71 suffixes and 241 short prefixes, leaving a lexicon of 559 suffixes and 1,462 prefixes.

As we will see in the Results section, the new approach did not improve the results. The precision obtained was 0.813 and the recall 0.747. That is, the precision did not improve, and there was a decrease in the recall.

## Results

As mentioned before, we divided SNOMED CT into hierarchies to simplify the translation process. We evaluated the *Clinical finding/disorder*, *Procedure *and *Body Structure *hierarchies, as they are the most populated ones. Since the *Clinical finding/disorder *hierarchy is specially populated we split it according to its semantic tags: *disorders *and *findings*.

### Phase 1 results

We want to remark that Phase 1 could not be evaluated in terms of the *quality *of the translations, but of *quantity*. As we used manually generated and checked dictionaries written by lexicographers and domain experts, we assumed the quality of the Basque terms. In any case, Basque is a language in its standardization process and some orthographic rules have been changed, so, the orthographic correctness of the descriptions and its possible disambiguation will be manually checked in the future.

Table 5 shows the evaluation of the Phase 1 regarding the quantities obtained from the different terminology resources. We distinguish the quantity of Basque equivalent terms obtained (column labeled as "#Syn.") and the number of source SNOMED CT concepts translated (column labeled as "#Concepts"). As seen in the table, the same concept may have more than one synonym. For instance, in the *Disorder *sub-hierarchy we have 3,063 SNOMED CT concepts translated and 3,975 Basque terms for the same concepts.

**Table 5:** Results of the Phase 1.

	English		Spanish		Total	
	
	**#Syn**.	#Concepts	**#Syn**.	#Concepts	**#Syn**.	#Concepts
**Disorder**	3,975	3,063	2,231	1,602	4,362	3,275
**Finding**	1,690	857	1,866	759	2,855	1,018
**Body Structure**	5,554	2,747	5,076	2,616	7,077	3,295
**Procedure**	557	405	536	377	775	501

If we consider the Total columns of the table (columns 6 and 7), we can observe that the totals do not match the sum of the previous columns. This is caused by the fact that the same equivalent term may be obtained from the English matching as well as from the Spanish matching, but it is counted only once. For example, the term "drepanozito" is obtained from the source term in Spanish *"drepanocito" *and from the English term "drepanocyte". This equivalence will be counted in both English and Spanish columns, but once in the Total columns.

We can highlight the amount of synonyms obtained in this Phase: 1.86 for each concept. *Body Structure *and *Disorder *hierarchies get the best results in terms of concepts translated (3,295 and 3,275 respectively), but it is remarkable the high amount of synonyms that *Body Structure *has (7,077 synonyms) which can be put down to the very specialized dictionaries devoted to this hierarchies: the Glossary of Anatomy and the ICD-10.

### Phase 2 results

In this phase, results are given for the simple terms extracted from the *Disorder*, *Finding*, *Body Structure *and *Procedure *hierarchies. The set of terms from each hierarchy is split into two: i) to define and develop the system and ii) to evaluate it.

The development and test sets comprise the simple terms that have been previously translated in the first phase of the algorithm. That is, we used the correct English-Basque pairs from the dictionaries as Gold Standard. This Gold Standard was manually created by setting a label to each term indicating whether or not the term should be translated by means of this system. That is, the system should not work with terms like "shock" or "dengue" that are not composed of medical roots.

For the evaluation set we took 848 terms from the *Disorder *sub-hierarchy, 375 from *Finding*, 774 from *Body Structure *and 248 from *Procedure*. The remaining 3,114 terms from *Disorder*, 1,446 from *Finding*, 1,838 from *Body Structure *and 1,729 from *Procedure *were used for development.

To measure the results of the experiment True Positives (TP), False Negatives (FN), False Positives (FP) and True Negatives (TN) are defined in the following way:

• True Positives: The term should be translated, it is translated and the translation is correct. That is, at least one of the Basque terms generated matches at least one synonym from the Gold Standard.

• False Negatives: The term should be translated and it is not translated.

• False Positives: The term should not be translated and it is translated, or the term should be translated and the Basque term generated is not correct.

• True Negatives: The term should not be translated and it is not translated.

Table 6 shows the precision, recall and F-Measure of the three approaches detailed in the Methods section. It is worth to mention that we obtain the best results regarding the F-Measure with the first approach. Even if the second approach gives a better precision compared to the first approach, the decrease it generates in the recall is much sharper, and so it is manifested in the F-Measure. Thus, we conclude that the best approach is the first one, and this is the one we use for the evaluation of the whole algorithm.

**Table 6:** Results of the Phase 2

		TP	FN	FP	TN	Total	**Prec**.	Recall	F-M
**Disorder**	**Baseline**	289	451	31	77	848	0.903	0.391	0.545
	**1st approach**	615	67	108	58	848	0.851	0.902	0.875
	**2nd approach**	577	104	102	65	848	0.850	0.847	0.849

**Finding**	**Baseline**	79	171	9	116	375	0.898	0.316	0.467
	**1st approach**	213	29	41	92	375	0.839	0.880	0.859
	**2nd approach**	178	63	32	102	375	0.848	0.739	0.789

**Body Structure**	**Baseline**	121	425	23	205	774	0.840	0.222	0.351
	**1st approach**	322	174	100	178	774	0.763	0.649	0.702
	**2nd approach**	284	212	91	187	774	0.757	0.573	0.652

**Procedure**	**Baseline**	98	77	9	64	248	0.916	0.560	0.695
	**1st approach**	144	16	49	39	248	0.746	0.900	0.816
	**2nd approach**	154	5	50	39	248	0.755	0.969	0.848

**Total**	**Baseline**	587	1,124	72	462	2,245	0.891	0.343	0.495
	**1st approach**	1,295	286	297	367	2,245	0.813	0.826	0.820
	**2nd approach**	1,304	275	299	367	2,245	0.813	0.747	0.779

We must consider that our evaluation does not take into account whether the system overproduces wrong Basque terms if the correct one is also produced. In any case, the overproduction is properly controlled as mentioned in the Methods section, and in average 1.05 Basque equivalents are produced from an English term.

Considering the results obtained in the Baseline approach, the changes made to the system in the first and in the second approaches show a huge improvement of the system. Even if we obtain a small decrease in the precision, the improvement in the recall is remarkable: changes from 0.343 in the baseline to 0.826 in the first approach and 0.747 in the second approach.

We must highlight that we focused the development of the system on the *Disorder *hierarchy as it is the one with more simple terms composed of Latin and Greek roots and affixes. The bias to this sub-hierarchy is evident as the *Disorder *sub-hierarchy obtains the best results.

### Overall results

We show the overall results of the translation algorithm in Table 7 regarding the mapping with the ICD-10 classification and the two phases implemented. That is, the table shows the synonyms obtained (named "#Syn." in the table) from the matches ("#Match" in the table) over the ICD-10 mapping, dictionaries and morphosemantics system. The "#Match" columns indicate the number of source terms translated, while the "#Syn." columns show the number of terms obtained. Remember that more than one term could be obtained from a unique source term.

**Table 7:** Results of the translation algorithm.

	Phase 0 ICD-10 mapping		Phase 1 Lexical resources		Phase 2 Morphosemantics		Total	
	**#Syn**.	**#Match**	**#Syn**.	**#Match**	**#Syn**.	**#Match**	**#Syn**.	**#Match**

**Disorder**	11,224	11,224	4,362	5,029	2,699	2,417	17,912	18,670
**Finding**	1,871	1,871	2,855	1,771	897	655	5,508	4,297
**Body Structure**	0	0	7,077	5,843	1,026	861	8,036	6,704
**Procedure**	0	0	536	835	1,780	1,427	2,490	2,262

The results labeled as "Phase 0 - ICD-10 mapping" in Table 7 show that the mapping is only relevant in the *Clinical disorder/finding *hierarchy and that the *disorder *semantic tag is the most benefited with 11,224 equivalences. In this case, the mapping does not offer synonyms, but obtains a single term from each mapping.

Table 8 shows the results regarding the number of tokens of the original English descriptions that are included in the source SNOMED CT, and it does not make reference to the number of concepts. The row labeled as *Translated *shows the quantity of English terms for which a translation has been obtained. The second row labeled as *Total *reveals the total amount of English terms, and finally, the last row presents the percentage of the translated terms.

**Table 8:** Results of the translation regarding the number of tokens of the original English term.

		1 token	2 tokens	3 tokens	4 tokens	>4 tokens	Total
**Disorder**	**Translated**	3,388	1,098	533	275	419	5,713
	**Total**	3,962	21,830	24,054	20,357	39,501	109,704
	**Percentage**	85.51%	5.03%	2.22%	1.35%	1.06%	5.21%

**Finding**	**Translated**	1,290	161	39	19	56	1,565
	**Total**	1,821	8,850	11,126	10,092	19,689	51,578
	**Percentage**	70.84%	1.82%	0.35%	0.19%	0.28%	3.03%

**Body Structure**	**Translated**	1,931	1,460	381	72	15	3,859
	**Total**	2,612	11,287	12,443	10,793	21,515	58,650
	**Percentage**	73.93%	12.94%	3.06%	0.67%	0.07%	6.58%

**Procedure**	**Translated**	1,741	80	11	2	1	1,835
	**Total**	1,982	9,966	15,848	16,578	37,695	82,069
	**Percentage**	87.84%	0.80%	0.07%	0.01%	0.003%	2.24%

The mentioned Table 8 is useful to measure the progress of the algorithm. That is, the first two phases of the algorithm are focused on single terms, whereas the remaining phases are designed for complex terms. We observe that a high percentage of the single terms is already translated in all the hierarchies, but specially in the hierarchies *Disorder *and *Procedure *(85.51% and 87.84% respectively). It is remarkable that 12.94% of the two tokens terms from *Body Structure *have already been translated from the dictionaries.

In order to give a wider view of the process followed, Table 9 presents the overall numbers of the translated SNOMED CT concepts.

**Table 9:** Overall results.

	Disorder	Finding	Body Structure	Procedure
**Translated Concepts**	14,181	2,953	3,845	1,607
**Concepts in total**	66,239	33,573	30,589	53,629
**Percentage**	21.41%	8.80%	12.57%	3.00%

Let us highlight the most promising results for each hierarchy:

• Regarding the *Disorder *sub-hierarchy, we obtained the translation of 21.41% of the terms (see Table 9). Considering that we have focused our work until now mainly on simple terms, we can consider that it is a very good result. The ICD-10 mapping contribution is the major one, producing 11,224 synonyms. In any case, the strength of the morphosemantics phase is noticeable in Table 8 which shows that 85.51% of the simple terms are translated.

• In regards to the *Finding *sub-hierarchy, we can consider it as the most balanced one, as it does not outline any method used. In this case, we achieved the translation of 8.80% of the concepts.

• In the *Body Structure *hierarchy, 12.57% of the concepts get a Basque equivalent, with outstanding results for complex terms (12.94% of two token terms).

• For the *Procedure *hierarchy the dictionaries are of hardly any use (536 Basque terms as seen in Table 7). In contrast, after applying the mophosemantics phase 87.84% of the simple terms are translated (see Table 8). In any case, we only obtain 3.00% of the concepts translated, and this must be an aspect to be improved in the following phases.

• In general, even if the overall numbers seems to be low (22,586 concepts translated over 184,030), it is a solid base to implement the following two phases in an incremental strategy.

## Conclusions

In this paper we presented some steps of an algorithm for the translation of the multilingual terminology content of SNOMED CT. We also described the good results obtained on the morphosemantics phase by means of an experiment, and how this phase and the dictionaries contribute on the translation of SNOMED CT by means of quantities.

On the one hand, we take advantage of existing lexical resources, and on the other hand, we use transducers to generate Basque equivalents by means of domain-specific affixes and transcription rules. The implementation can be available on request contacting the authors. It will be publicly accessible once the implementation is concluded.

Even if the specialized dictionaries provide Basque simple and complex terms, in this case the transducers are designed to translate simple terms. Thus, we got the translation of 85.51% of the simple terms in the *Disorder *sub-hierarchy and 87.84% in the *Procedure *hierarchy.

Even if in this paper we only show the results obtained in the most populated hierarchies, we applied the translation algorithm to the whole SNOMED CT terminology. The use of lexical resources is promising as seen in the Results section, and the contribution of the ICD-10 mapping in the *Disorder *sub-hierarchy is especially remarkable (11,224 matchings). The *Disorder *sub-hierarchy is the largest and here we obtained the equivalents in Basque of 5.21% of the source English terms.

Nevertheless, as we said before, our aim is to check the quality of the Basque SNOMED CT version we are generating. For this evaluation (and correction) we count on the help of specialists of medical terminology such as doctors and terminologists. We consider the *linguistic correctness *of the translation and the *fidelity of the translated content *are appropriate for this evaluation of the translation quality. In addition, we are working in a platform to help the specialists with the evaluation and correction. If the quality of the terminology generated reaches high and solid results, we will contact the SNOMED CT providers to offer them the result of our work, which at the moment is in the field of academic research.

In regard to the evaluation of our systems, the first phase does not require a deep evaluation as it extracts English-Basque and Spanish-Basque pairs from dictionaries. In any case, a deeper evaluation of the approaches based on morphosemantics is presented. We implemented and evaluated three systems for the translation of simple terms using morphosemantic characteristics of the terms.

In the future, we plan to implement the remainder of the algorithm in two ways: on the one hand, to generate the complex terms by means of syntax rules and on the other hand, to adapt the machine translation tool. The promising results obtained up to the present encouraged us to finish the semi-automatically generated version in Basque of SNOMED CT.

## List of abbreviations used

SNOMED CT: Systematized Nomenclature of Medicine - Clinical Terms. HIT: Health Information Technology. EHR: Electronic Health Record. FSN: Fully Specified Name. PT: Preferred Term. ICD-10: International Statistical Classification of Diseases and Related Health in its 10th version. True Positive: TP. False Positive: FP. False Negative: FN. True Negative: TN. FST: Finite State Transducer.

## Competing interests

The authors declare that they have no competing interests.

## Authors' contributions

OPV performed the implementation of the algorithm. OPV and MO wrote, read and approved the final manuscript.

## Declarations

Publication costs for this article were funded by the Basque Government, project number IT344-10 (IXA group, Research Group of type A).

This article has been published as part of *BMC Medical Informatics and Decision Making *Volume 15 Supplement 2, 2015: Proceedings of Louhi 2014: The Fifth International Workshop on Health Text Mining and Information Analysis. The full contents of the supplement are available online at http://www.biomedcentral.com/bmcmedinformdecismak/supplements/15/S2.
